# *Staphylococcus aureus* Cell Wall Phenotypic Changes Associated with Biofilm Maturation and Water Availability: A Key Contributing Factor for Chlorine Resistance

**DOI:** 10.3390/ijms24054983

**Published:** 2023-03-05

**Authors:** Farhana Parvin, Md. Arifur Rahman, Anand K. Deva, Karen Vickery, Honghua Hu

**Affiliations:** 1 Macquarie Medical School, Macquarie University, Sydney 2109, Australia; 2 Innovation Centre of Translational Pharmacy, Jinhua Institute of Zhejiang University, Jinhua 321016, China

**Keywords:** *Staphylococcus aureus*, biofilm, cell wall, peptidoglycan, biocide resistance, disinfectant tolerance

## Abstract

*Staphylococcus aureus* biofilms are resistant to both antibiotics and disinfectants. As *Staphylococci* cell walls are an important defence mechanism, we sought to examine changes to the bacterial cell wall under different growth conditions. Cell walls of *S. aureus* grown as 3-day hydrated biofilm, 12-day hydrated biofilm, and 12-day dry surface biofilm (DSB) were compared to cell walls of planktonic organisms. Additionally, proteomic analysis using high-throughput tandem mass tag-based mass spectrometry was performed. Proteins involved in cell wall synthesis in biofilms were upregulated in comparison to planktonic growth. Bacterial cell wall width (measured by transmission electron microscopy) and peptidoglycan production (detected using a silkworm larva plasma system) increased with biofilm culture duration (*p* < 0.001) and dehydration (*p* = 0.002). Similarly, disinfectant tolerance was greatest in DSB, followed by 12-day hydrated biofilm and then 3-day biofilm, and it was least in the planktonic bacteria––suggesting that changes to the cell wall may be a key factor for *S. aureus* biofilm biocide resistance. Our findings shed light on possible new targets to combat biofilm-related infections and hospital dry surface biofilms.

## 1. Introduction

Biofilms are composed of a combination of sessile communities of microbial cells and a substratum or interface. The cells are encased in a matrix of extracellular polymeric substances (EPS) [1]. Biofilms have an adaptive advantage over planktonic organisms as bacteria within biofilm are protected from harsh environmental conditions. Biofilm bacteria have been shown to withstand extreme temperatures and pH, desiccation, antimicrobial treatment, and nutrient scarcity, as well as mechanical stresses such as high pressure and shear forces [1,2,3,4].

There are several biofilm variants, including traditional hydrated biofilm that is formed in the presence of water [1], build-up-biofilm (BUB) [5], and dry surface biofilm (DSB) [6]. BUB forms on medical instruments subjected to cycles of patient use (contamination and bacterial growth), intense cleaning, high-level disinfection/sterilisation (physical and chemical stress), and storage (water stress). DSB forms on dry hospital surfaces and is subjected to water stress. DSBs formed on hard surfaces are also subjected to infrequent (at most, daily) cleaning and disinfection (hand pressure and low-level disinfection using hospital-grade disinfectants). Worldwide, DSB contaminates between 70 and 95% of dry hospital surfaces and most contain antibiotic-resistant organisms, including methicillin-resistant *Staphylococcus aureus* (MRSA) [3,7,8,9].

Many studies have confirmed that hydrated biofilms are more resistant to disinfectants than planktonic organisms [10]. DSB is even more difficult to kill than hydrated biofilm, surviving up to 20 times the normal concentration of sodium hypochlorite [11]. Contamination of dry hospital surfaces emphasises the failures of current decontamination practices, including reduced efficacy of disinfectants [10,11,12] and the difficulty in removing DSB by cleaning methods that are normally used [13,14]. The mechanisms proposed for biofilm tolerance of antimicrobials include decreased diffusion into the biofilm, biocide inactivation by the EPS, bacterial stress response, upregulated efflux pumps, and the physiology of the embedded cells, including a lower metabolic rate [2]. We hypothesised that *S. aureus* subjected to periodic water stress will form a biofilm in which the bacterial cell wall is thickened to reduce water loss from the cell, akin to the thickened cell walls present in spores. Furthermore, it is this adaption that is a principal component of DSB’s ability to withstand harsh environmental conditions, including tolerance to desiccation and disinfectants.

For this study, we aimed to focus *S. aureus* biofilms, a frequent cause of healthcare-associated infections (HAI) [15]. Staphylococci are incorporated in over 80% of clinical DSB, and between 50% and 58% contain pathogenic *S. aureus*, embedded in very thick EPS [3,7]. Despite being embedded in thick EPS, *S. aureus* DSB can be easily transmitted by the hands to other surfaces [16]. In addition, *S. aureus* contamination of healthcare workers’ hands occurs more frequently from the environment than from patients [17]. Additionally, the principal bacteria isolated from biofilms around medical devices are *S. aureus* and coagulase-negative staphylococci [18,19], leading in many cases to medical device failure and the need for removal [20].

The bacterial cell wall provides structural integrity and is important for bacterial cell survival and growth. Furthermore, the cell wall acts as a major line of defence against toxins, including antimicrobials [21]. The key element and structural scaffold of Gram-positive bacterial cell walls is peptidoglycan, which is composed of a glycan chain linked by short peptide bridges to form a continuous network that surrounds the cell. Other cell wall components, such as capsular polysaccharides, teichoic acid, and proteins are covalently attached to this scaffold [21,22]. As a result of its importance, the cell wall is a target for several antibiotics that interfere with cell wall synthesis and cause cell wall structural and functional defects. Several studies have revealed increased cell wall thickness is characteristic of vancomycin and daptomycin resistance in *S. aureus* [23,24].

In this study, we investigated phenotypic characteristics of *S. aureus* cell walls when grown as planktonic organisms, 3-day hydrated biofilm, 12-day hydrated biofilm, and 12-day DSB, and we related these characteristics to disinfectant tolerance. In addition, we used high-throughput tandem mass tag (TMT)-based mass spectrometry (MS) to examine changes in protein expression profiles found in differing biofilms. Morphological and proteomic studies may lead to a better understanding of bacterial antimicrobial tolerance mechanisms. Additionally, proteomic studies may shed light on possible antimicrobial targets common to all *S. aureus* biofilms.

## 2. Results

### 2.1. Proteomics of Biofilms

**Figure 1:**
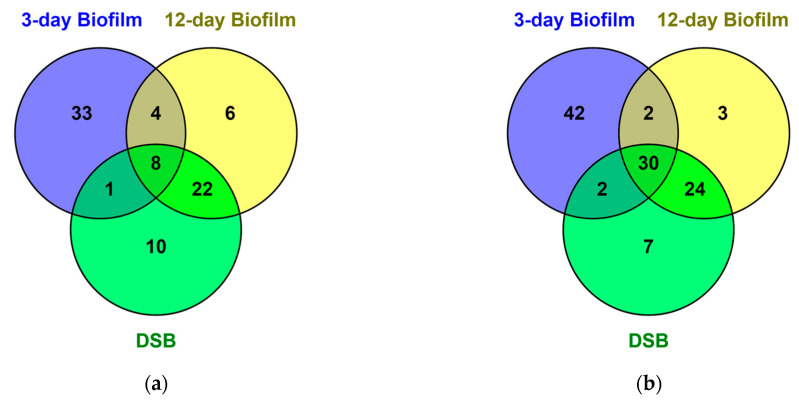
This Venn diagram represents identified proteins dysregulated >2-fold, *p* < 0.05 in comparison with 24 h planktonic culture. Proteins significantly upregulated (**a**) or downregulated (**b**) in 3-day hydrated, 12-day hydrated, and dry surface biofilm (DSB).

We identified and quantitated 1636 non-redundant proteins with at least one unique peptide and <1% FDR. Of these, 194 proteins were found in all three biofilms and differentially regulated in biofilm compared to planktonic organisms (cut-off range > 2-fold, *p* < 0.05) (Figure 1). Eighty-four proteins were upregulated in biofilm and 110 were downregulated. Of these, 8 proteins were upregulated, and 30 proteins were downregulated in all three biofilms compared to planktonic culture (Figure 1, Supplementary Tables S1 and S2). Additional upregulated and downregulated proteins occurring in only one biofilm growth condition or in two of the three different biofilms are listed in Supplementary Tables S3–S6.

Upregulated proteins common to all biofilms included those involved in energy metabolism, amino acid biosynthesis, and metabolism, such as 2-oxoisovalerate dehydrogenase, which was upregulated 3.19-fold in hydrated 3-day biofilm, 2.12-fold in 12-day hydrated biofilm, and 2.06-fold in DSB. 2-oxoisovalerate dehydrogenase is involved in multiple biosynthesis and metabolic pathways, including amino acid and central carbon and energy metabolism (e.g., TCA cycle) (Supplementary Table S1). Four proteins associated with purine metabolism were upregulated, including guanosine 5′ monophosphate oxidoreductase, upregulated 2.28, 3.28, and 2.97-fold in 3-day, 12-day hydrated biofilm, and DSB, respectively (Supplementary Table S1).

D-alanine transfer protein DltB, is important for cell wall teichoic acid modification, and was upregulated 2.1-fold in the 3-day biofilm but over 3-fold in both the older 12-day biofilm and DSB.

Four proteins common to all three biofilms were only upregulated in hydrated biofilm, including glutamine-fructose-6-phosphate transaminase isomerizing (GlmS), while 22 proteins were upregulated only in the older 12-day hydrated and 12-day DSB. These included 2-oxoisoalerate dehydrogenase E1 component upregulated over 3-fold and three proteins (carbamoyl-phosphate synthase small chain, cytidine triphosphate synthase, and orotate phosphoribosyl transferase) involved in metabolic pathways, and pyrimidine metabolism were upregulated over 2-fold (Figure 1).

There were 30 proteins significantly downregulated >2-fold in all three biofilms compared to planktonic bacteria. Downregulated proteins occurred in the categories of pyruvate metabolism, energy metabolism, amino acid biosynthesis, and translation indicating a lower metabolic activity profile in biofilm (Supplementary Table S2). An additional 24 proteins were downregulated in older biofilm compared to planktonic bacteria and 3-day biofilm (Supplementary Table S6).

### 2.2. Scanning Electron Microscopy

**Figure 2:**
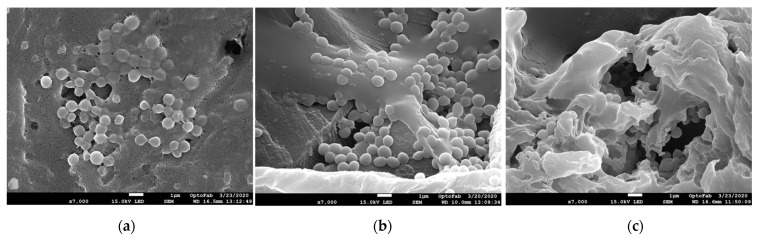
Scanning electronic micrographs of *S. aureus* biofilm. (**a**) 3-day hydrated biofilm; (**b**) 12-day hydrated biofilm; (**c**) 12-day DSB biofilm. Scale bar on the images represent 1 µm with 7000× magnification.

Scanning electron microscopy (SEM) confirmed the formation of bacterial biofilms on polycarbonate coupons. All three biofilms were multi-layered and formed a continuous coating on the coupon. However, there were clearly identifiable differences between the different biofilm in the amount of EPS present. The 3-day hydrated biofilm had the least EPS (Figure 2a) and the DSB had the most. While many of the cells were embedded in the EPS, it was still easy to see individual cells sitting on top of the EPS surface or partly covered by the EPS in both the 3-day and 12-day hydrated biofilm (Figure 2a,b). In contrast, the EPS matrix was densest in the DSB and it was difficult to discern individual cocci (Figure 2c).

### 2.3. Transmission Electron Microscopy

**Figure 3:**
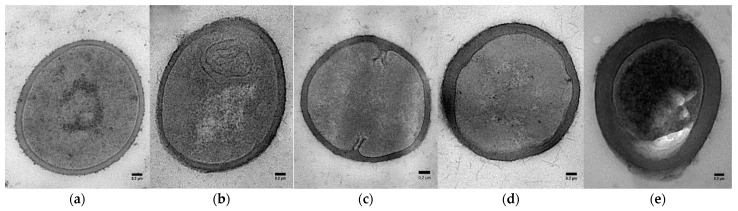
TEM micrographs (**a**) 1-day planktonic (1DP); (**b**) 3-day planktonic (3DP); (**c**) 3-day wet biofilm (3DB); (**d**) 12-day wet biofilm (12DB); (**e**) 12-day dry biofilm (DSB). Scale bar on the images represent 0.2 µm with 46,000× magnification.

**Figure 4:**
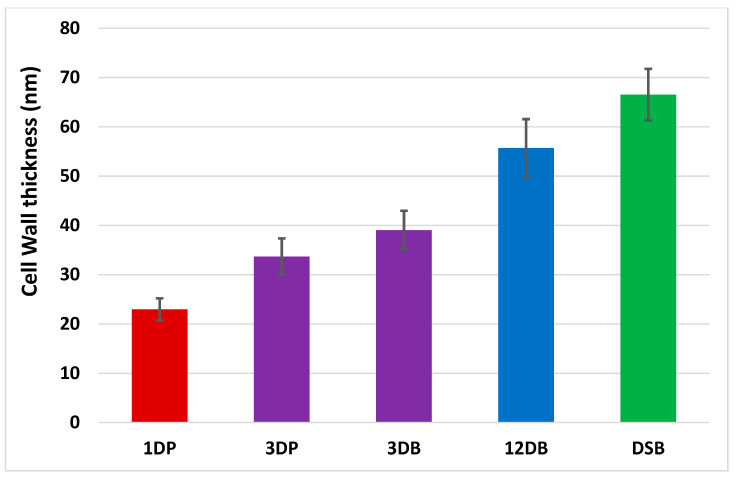
Cell wall thickness of *S. aureus* strain grown using different culture conditions. Columns of different colours pairwise comparisons were significantly different from each other (*p* < 0.001 for all except DSB and 12-day hydrated biofilm *p* = 0.002). Only 3-day planktonic and 3-day biofilm cell walls (columns of the same colour) were not significantly different from each other.

A comparison of the transmission electron microscopy (TEM) images (Figure 3) demonstrated that cell wall thickness significantly increases with culture duration for both planktonic (one-day versus 3-day, *p* < 0.001) and biofilm cells (3-day versus 12-day hydrated biofilm and 12-day DSB (*p* < 0.001) (Figure 4). However, although 3-day hydrated biofilm bacteria had thicker cell walls (39.03 ± 3.93 nm) in comparison to 3-day planktonic cells (33.67 ± 3.69 nm), the difference was not significant (Figure 4). Water restriction during biofilm production affected the cell wall thickness, with 12-day DSB (66.52 ± 5.24 nm) having significantly thicker cell walls than 12-day hydrated biofilm (55.69 ± 5.86 nm) (*p* = 0.002). The cell walls of bacteria incorporated into DSB were 2.9 times thicker, 12-day hydrated biofilm 2.4 times thicker, 3-day hydrated biofilm 1.7 times thicker, and 3-day planktonic 1.5 times thicker than 1-day planktonic cell walls (*p* < 0.001) (Figure 4).

The maximum cell size was variable with planktonic cells being an average of 0.318 ± 0.05 nm (range 0.225–0.377 nm). Variability was particularly evident for the 3-day planktonic culture (mean cell size 0.392 ± 0.1 nm) but with some very large cells (0.646 nm) and some small cells (0.245 nm). The 3-day (mean 0.281 ± 0.05) and 12-day hydrated biofilm (mean 0.281 ± 0.06) were of similar size to each other and not significantly different from the planktonic cells. The DSB cells (mean 0.214 ± 0.04 nm) were on average smaller than all the other culture cells and only 70% of the size of one-day planktonic cells (*p* < 0.05).

### 2.4. Peptidoglycan Measurement

The average number of bacteria from each DSB-covered coupon was log_10_ 7.095 ± 0.14, whereas log_10_ 6.53 ± 0.19 and log_10_ 7.00 ± 0.16 bacteria grew from each 3-day and 12-day hydrated biofilm coupon, respectively. The number of bacteria in the biofilm significantly increased from 3 to 12 days (*p* < 0.001). There was no significant difference in the number of bacteria in 12-day hydrated or 12-day DSB. These figures were used to normalise results for an equal number of bacteria for peptidoglycan measurements.

**Figure 5:**
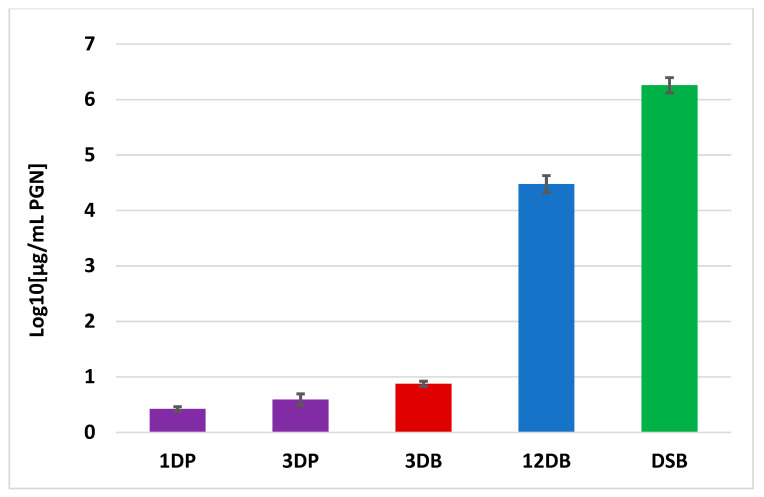
Peptidoglycan concentration of number *S. aureus* cells grown in planktonic and biofilm mode. [Bars of different colour are significantly different from each other (*p* < 0.05)].

There was significantly more peptidoglycan in biofilm samples than in one-day planktonic bacteria. The maximum amount of peptidoglycan was found in DSB and this was significantly more than samples in other growth conditions (*p* < 0.001). The 12-day hydrated biofilm had significantly more peptidoglycan than the 3-day hydrated biofilm, and both 1- and 3-day planktonic cells (*p* < 0.001). The 3-day hydrated biofilm sample had significantly more peptidoglycan than one-day planktonic growth (*p* = 0.003) and 3-day planktonic samples (*p* < 0.05). Although there was more peptidoglycan in 3-day planktonic samples, this was not significantly different from 1-day planktonic samples (Figure 5).

### 2.5. Disinfectant Efficacy Testing Results

The average number of bacteria in the positive controls (n = 5) was log_10_ 6.96 ± 0.03, log_10_ 7.16 ± 0.17, log_10_ 7.23 ± 0.13, and log_10_ 7.05 ± 0.11 for 1-day planktonic, 3-day biofilm, 12-day biofilm, and DSB. All negative controls were negative, 20% bovine serum albumin was non-toxic to *S. aureus*, and it was able to neutralise the activity of sodium hypochlorite when made up to give an available chlorine concentration of up to 5000 mg/L (results not shown).

**Figure 6:**
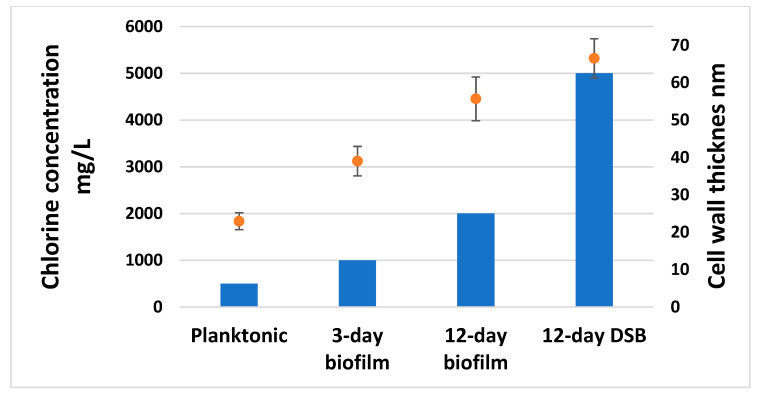
The concentration of available chlorine (column graph) necessary to kill 10^7^ *S. aureus* planktonic, hydrated 3-day, and 12-day biofilm, and dry surface biofilm cells (DSB) and corresponding cell wall thicknesses (mean ± std) (orange dot and bar).

**Figure 7:**
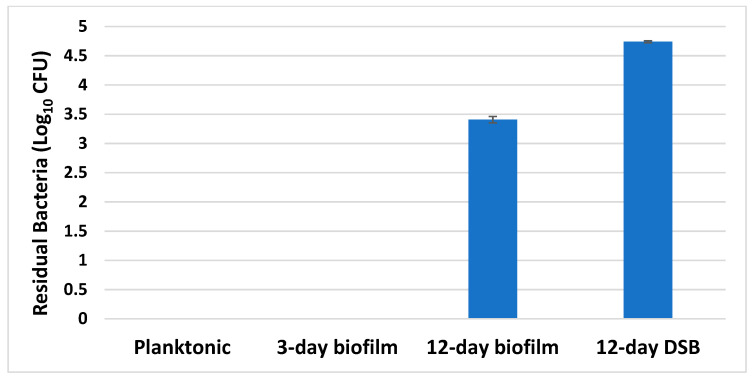
Number of viable bacteria remaining (mean ± std) following treatment with 1000 ppm available chlorine on approximately 10^7^ planktonic, 3-day biofilm, 12-day biofilm, and DSB *S. aureus*.

The concentration of available chlorine necessary to kill mature hydrated 12-day biofilm cells and DSB was much higher than that required to kill planktonic cells (Figure 6 and Supplementary Table S7). Healthcare facilities commonly use a concentration of 0.1% (1000 mg/L) to disinfect hard surfaces. This concentration killed both the planktonic and 3-day biofilm, but it was unable to kill more mature biofilm or DSB, leaving large numbers of viable cells (Figure 7).

## 3. Discussion

Our findings confirmed that 1000 mg/L free chlorine was able to eradicate 7 Log_10_ planktonic and 3-day biofilm cells. By comparison, 12-day-old biofilm required twice the concentration of free chlorine to achieve eradication. Subjecting the biofilm to periodic dehydration increased the disinfectant tolerance even further, with DSB requiring 5000 mg/L concentration of free chlorine or ten times the concentration needed to kill the same number of planktonic organisms. The higher concentration of disinfectant required to kill older biofilm and DSB has direct implications for infection control in the healthcare setting. Current use of 0.1% (1000 mg/L) free chlorine to disinfect hard surfaces may not be effective in eradicating established DSB and may be ineffective in reducing the risk of HAI. From our data, established and mature biofilm also failed to be eradicated with the standard 1000 mg/L (Figure 7). We have previously shown that very high concentrations of sodium hypochlorite (20,000 mg/L) failed to fully eradicate *S. aureus* DSB, leaving viable but non-culturable (VBNC) bacterial cells after treatment. We were not able to culture live bacteria post-treatment, but confocal microscopy in conjunction with Live/Dead staining showed that while most of the biofilm mass was removed with only 1.4% of biofilm cells remaining, 60% of these cells were alive. Upon prolonged culture, the cells were able to reconstitute the biofilm and eventually release planktonic cells into the media [11]. DSB was found significantly harder to kill than hydrated biofilm [12,14].

There might be VBNC cells present after chlorine treatment in this study. We used horse blood agar plates to recover bacteria after chlorine treatment. Horse blood agar plate is considered suitable for isolating most pathogens but isn’t considered really rich, won’t isolate fastidious organisms, and may not be able to isolate VBNC cells. Using enriched media, cells that are in the VBNC state on less enriched media could be induced to grow. However, a state may be reached when even enriched media falls to induce VBNC cells to grow. In a future study, we could investigate different ways to induce the VBNC cells to grow and eradicate these VBNC cells as well.

The cell wall is a major line of defence for bacteria, and increased cell wall thickness imparts resistance to some antibiotics. TEM of clinical isolates of methicillin-resistant *S. aureus* have significantly thickened cell walls and septum compared with methicillin-sensitive strains (*p* < 0.001) [25]. Both vancomycin and daptomycin resistance in *S. aureus* is associated with increased cell wall thickness and changes in the cell wall teichoic acids [23,24]. Furthermore, the degree of cell wall thickening was found to correlate with the MIC of vancomycin [26]. Our study found that increased disinfectant tolerance corresponded to increased cell wall thickness, with cell wall phenotypic changes being related to both culture age and culture conditions. As planktonic cultures aged, the cell walls became significantly thicker between days 1 and 3 (*p* < 0.001). There was no significant difference in mean cell wall thickness between 3-day planktonic and 3-day biofilm, although the 3-day planktonic cells were highly pleomorphic. Biofilm cell wall thickness increased significantly between days 3 and 12 (*p* < 0.001) in the absence of osmotic stress, but the cell size remained the same. Longer biofilm culture time also resulted in significantly increased concentration of peptidoglycan (*p* < 0.001). However, subjecting biofilm to osmotic stress during culture induced the greatest increase in cell wall thickness and peptidoglycan concentration, with DSB having significantly thicker walls (*p* = 0.002) and more peptidoglycan (*p* < 0.001) than 12-day hydrated biofilm, despite the biofilms being the same age and the analysis being conducted on the same number of cells. Inducing osmotic stress by culturing *S. aureus* in 10% or 20% sodium chloride also results in increased cell wall thickness [27]. In addition to increasing cell wall thickness, the morphology and cell size of bacteria grown on typtic soya broth (TSB) agar changed, with decreased relative humidity at the culture/air interface as well [28]. With decreased water availability, Gram-negative species become filamentous and form wave-like patterns, sometimes twisting together, to reduce their exposed surface area to volume ratio [28]. These results were similar to results obtained by earlier workers utilising sodium chloride or glycerol, to reduce water availability, in liquid systems [29].

The peptidoglycan concentration is significantly greater in biofilm than in planktonic cells, even cells of the same age, i.e., 3-day hydrated biofilm compared to the 3-day planktonic cells, suggesting that the extra peptidoglycan was being deposited as EPS (*p* < 0.05). As the biofilm aged, the peptidoglycan concentration increased, but the greatest concentration of peptidoglycan was found in DSB subjected to osmotic stress. In this study, we found that GlmS, while present in all biofilms, was significantly upregulated in the hydrated biofilms. GlmS catalyses the reaction to produce glucosamine-6-phosphate from fructose-6-phosphate during the initial steps of peptidoglycan synthesis. It is also involved in metabolic pathways, including amino acid metabolism, nucleotide sugar metabolism, and biosynthesis of antibiotics, alanine, aspartate, and glutamate.

Similarly, our previous proteomic studies have shown that proteins involved in peptidoglycan biosynthesis are upregulated in biofilm compared to planktonic cells [30]. In 3-day biofilm, UDP-N-acetylglucosamine 1-carboxyvinyltransferase 1 (murA) and N-acetylmannosamine-6-phosphate 2-epimerase (namE) were upregulated [30]. murA adds enolpyruvyl to UDP-N-acetylglucosamine during peptidoglycan biosynthesis. NamE catalyses carbohydrate epimerisation converting N-acetylmannosamine-6-phosphate to N-acetylglucosamine-6-phosphate, which leads to peptidoglycan biosynthesis. In 12-day biofilm FemX, which catalyzes the incorporation of the first glycine of the pentaglycine interpeptide bridge of peptidoglycan, was upregulated [30]. While in DSB, UDP-N-acetylenolpyruvylglucosamine reductase (MurB) and UDP-N-acetylmuramic acid: L-alanine ligase (MurC) were upregulated. murB catalyses the second committed step in peptidoglycan biosynthesis, converting enolpyruvyl UDP-acetylglucosamine to UDP-N-acetylmuramic acid, to which MurC adds a L-alanine [31]. These findings were further confirmed by TEM. The increased cell wall thickness occurs in the absence of antibiotic selection, yet it may contribute to the antibiotic tolerance seen in *S. aureus* biofilms. We believe that these cell wall changes are an environmental adaptation rather than due to genetic mutation.

In addition to peptidoglycan, teichoic acid is an important cell wall component. The addition of D-alanine esters to the teichoic acids by D-alanine transfer proteins gives the bacterial cell wall an overall positive charge, thus increasing resistance to antimicrobials, such as cationic peptides [32]. D-alanine transfer protein DltB is thought to aid the transfer or increase the rate of transfer of D-alanine across the cell membrane [33]. In this study, we found that DltB was upregulated 2.1-fold in 3-day hydrated biofilm but over 3-fold in both the older 12-day hydrated and dehydrated biofilm, suggesting extensive D-alanine modification of all *S. aureus* biofilms. Without D-alanine modifications of teichoic acid, *S. aureus* attachment to surfaces and subsequent biofilm formation can be inhibited [34]. The upregulation of this protein in all three biofilms suggests that it is important in promoting biofilm formation and *S. aureus* cell wall modifications promoting biocide tolerance, and could be a candidate for anti-biofilm therapy.

Biofilms forming in water distribution systems are hydrated and contain about 10% dry matter consisting of cells and EPS while DSB are semi-dehydrated, containing about 61% dry matter and 39% water [4]. As shown in Figure 2, the EPS surrounding bacteria in the three classes of biofilm was visually different. Older *S. aureus* biofilm (48 versus 24 h biofilm) has previously been shown to have more EPS [35]. Similarly in this study, the older 12-day biofilm had more EPS than the 3-day biofilm. However, both the 3-day and 12-day hydrated biofilms had significantly less EPS than the DSB, which received intermittent nutrition separated by long periods of dehydration. As the DSB and 12-day hydrated biofilm contained the same number of cells but the DSB contained six times the dry matter, this suggests that the physiochemical environment of bacteria in DSB is very different from the physiochemical environment of cells in hydrated biofilm. Similarly, osmotic stress, induced by desiccation or the addition of either NaCl or glucose to TSB, increased the number of S. epidermidis clinical isolates that were able to form biofilm and/or increased the amount of biofilm formed [36,37]. EPS retains water, thus protecting the bacteria from dehydration [37]. Extra thick EPS is likely an adaption to growing on dry surfaces. We have shown that DSB sourced from dry hospital surfaces protects incorporated bacteria, including pathogens, from desiccation for over 12 months [3].

The penetration of some antimicrobial agents has been shown to be hindered by the EPS, and molecule size has been shown to be a factor in decreased diffusion, with larger particles taking longer [38]. However, reduced diffusion can result from binding and inactivation in the EPS, particularly with reactive chemicals. Using microelectrode technology, hypochlorite took 48 min to penetrate 1 mm thick biofilm while the less reactive chlorosulfamate formulations only took 6 min, despite the chlorosulfamate disinfectant having a larger molecular weight [39]. The resistance of Salmonella enterica serovar Typhimurium biofilm to disinifectant is thought to arise from a combination of decreased disinfectant diffusion through the EPS and upregulation of efflux pumps [40]. The biocide resistance of *S. aureus* DSB could also be related to the increased EPS retarding disinfectant perfusion or a direct inactivation of the disinfectant by EPS.

Components of hydrated staphylococcal biofilms include fibronectin-binding proteins (FnBPs), biofilm-associated proteins (Bap), polysaccharide intercellular adhesion (PIA), extracellular DNA (eDNA), and teichoic acids [41]. Although the components of DSB EPS matrix are unknown, our published DSB model was composed of 56% protein, 24% glycoconjugates, and 20% DNA [42]. eDNA is important in maintaining the integrity of the EPS and may be critical in preventing/retarding biofilm penetration and dispersal by biocides [43,44]. In this study, four proteins involved in purine metabolism, including guanosine 5′-monophosphate oxidoreductase, were upregulated in all three biofilms (Supplementary Table S1). The older biofilms had 22 proteins upregulated, including three proteins (carbamoyl-phosphate synthase small chain, triphosphate synthase, and orotate phosphoribosyl transferase) involved in pyrimidine metabolism. The upregulation of both purine and pyrimidine metabolism in older biofilm reflects the increased EPS surrounding the cells and suggests that DNA stabilisation of biofilm remains important irrespective of biofilm habitat. Additionally, 2-oxoisoalerate dehydrogenase E1 component was upregulated over 3-fold in the two older biofilms. This protein is involved in metabolic processes converting keto acids (deaminated amino acids) to acyl-CoA, and ultimately to acetyl-CoA and the TCA cycle, thereby providing a source of energy in older biofilm.

In our study, we do concede that variation in culture conditions leading to variation in nutrient accessibility and temperature of incubation between hydrated biofilm and DSB may potentially limit our findings. Hydrated biofilm was grown at a constant 37 °C, initially in 50% TSB, which was reduced to 20% TSB at 48 h and refreshed every 48 h. In contrast, DSB was grown in only 5% TSB to model the minimal nutrition available on dry hospital surfaces. The temperature of incubation varied from 35 °C during the nutritional phases to 22–25 °C during the dehydration phases.

In summary, peptidoglycan synthesis is upregulated in *S. aureus* biofilm and results in increased cell wall thickness and increased deposition of EPS. The cell wall becomes thicker as the biofilm ages and/or if the biofilm is subjected to periodic osmotic stress. In addition, D-alanine transfer protein DltB, which aids the D-alanine modification of teichoic acid is upregulated. These cell wall changes mimic those found in daptomycin-resistant *S. aureus* [24] due to genetic mutation, and they may contribute to antibiotic tolerance seen in *S. aureus* biofilms lacking genetic mutations. The tolerance of *S. aureus* to sodium hypochlorite was increased as peptidoglycan synthesis increased, which was reflected in increased cell wall width and increased EPS production. Although pathways and mechanisms leading to clinical DSB formation are unknown, our findings may trigger target-based approaches for designing novel chemical classes aimed at peptidoglycan synthesis to aid in the killing of biofilms.

Biofilms confer increased resistance to bacteria through a variety of postulated mechanisms including the inactivation of antibiotics by the EPS, increased efflux pumps, quorum sensing and persister cells. In this study, we have shown that as the biofilm ages the cell wall becomes thicker. Our findings suggest that phenotypic changes to the cell wall associated with biofilm formation is one of the major contributors to antibiotic/antiseptic resistance of biofilms.

DSB contaminate over 90% of hospital surfaces and can contain multidrug-resistant organisms such as MRSA. DSB are more tolerant of disinfectants and harder to physically remove than planktonic organisms and hydrated biofilm. The current practice of cleaning, disinfection, and decontamination are not sufficient to eradicate DSB and allow DSB to spread pathogenic bacteria resulting in HAI. In this study, we have shown that a thickened bacterial cell wall in DSB plays a major role in its resistance to both antibiotics and antiseptics. The development of novel compounds that target or are able to penetrate thickened bacterial cell walls to help eradicate DSB are needed.

## 4. Methods

### 4.1. Bacterial Culture

Planktonic *S. aureus* (ATCC 25923) was grown to stationary phase in 100% tryptone soy broth (TSB, Thermo Fisher Scientific, Waltham, MA, USA) for 24 h and 3 days at 37 °C and 130 rpm without changing the media.

Hydrated *S. aureus* biofilms were developed on sterile removable polycarbonate coupons using the CDC bioreactor (BioSurface Technologies Corporation, Bozeman, MT, USA) for 3 days or 12 days in the batch phase. The CDC bioreactor was inoculated with 10^8^ *S. aureus* cells in 500 mL of 50% TSB and incubated for 48 h at 37 °C with a constant baffle rotation of 130 rpm. Thereafter, the media was replaced with 20% TSB every 48 h.

DSB was grown in 5% TSB at 35 °C for 48 h, followed by 48 h of dehydration at room temperature (22–25 °C). A further three cycles of batch growth in 5% TSB for 6 h was alternated with extensive dehydration phases of 66, 42, and 66 h at room temperature, as described previously [42]. During batch phases, shear was provided by baffle rotation of 130 rpm. Throughout the biofilm culture, filter-sterilised room air was pumped into the bioreactor at 3 L/min.

### 4.2. Colony Forming Units Determination

Biofilm-covered coupons were separated from the generator and rinsed three times in phosphate-buffered saline (PBS) to remove loosely attached bacteria. Coupons were then subjected to sonication in an ultrasonic bath (Soniclean, JMR, Sydney, Australia) at 42–47 kHz for 20 min, followed by vertexing for 2 min before sequential 10-fold dilution and standard plate culture on horse blood agar plates at 37 °C for 48 h and colony forming unit (CFU) determination.

### 4.3. Proteomics Analysis of Biofilm

Proteomics analysis of the *S. aureus* biofilm was performed according to our published methods [30,31]. In summary, we conducted protein extraction and fractionation, reduction, alkylation, and in-solution digestion, and performed TMT-based high-throughput mass spectrometry (MS) of planktonic, mature (3 days), and prolonged (12-day) biofilms, and 12-day dry surface biofilms (DSB). Planktonic bacteria were pooled from three independent growths of 24 h *S. aureus* cultures and used as a control in MS analysis. Bacterial culture conditions were the same as described in Section 4.1. In addition, the identified potential marker proteins were subjected to pathway analysis, subcellular localization, and protein-protein interaction (PPI) network mapping [30,31]. For detailed methods, please see Supplementary Methods.

### 4.4. Scanning Electron Microscopy

Biofilm-covered coupons were washed with PBS three times to remove loosely attached bacteria, fixed in 3% glutaraldehyde, and dehydrated through alcohol and hexamethyldisilazane (HMDS, Polysciences Inc., Warrington, PA, USA) before the sputter coating with 20 nm gold film, as described previously [45], and examined in a scanning electron microscope (JEOL JSM 7100F Field Emission Scanning Electron Microscopes (FESEM), Japan Electron Optics Laboratory, Tokyo, Japan).

### 4.5. Transmission Electron Microscopy

Planktonic bacterial cells were pelleted by centrifugation at 5000 rpm for 5 min whereas biofilms were scraped off coupons and pelleted by centrifugation at 7000 rpm for 2 min. Cell pellets were added to liquid 4% low melting point agarose (Sigma-Aldrich, St. Louis, MO, USA), spun for 30 s at 14,000 rpm, and cooled at 4 °C to solidify the agarose. The agarose block was sectioned into 1–2 mm pieces and washed in PBS three times for 5 min before fixing in a mixture of 2.5% glutaraldehyde and 3% paraformaldehyde at room temperature for 1 h followed by washing in PBS three times for 5 min. Samples were post-fixed in 1% osmium tetraoxide in PBS for 1 h and re-washed. Samples were dehydrated through ethanol, infiltrated with LR White resin (Agar Scientific, Stansted, UK), and polymerized in gelatine capsules at 65 °C overnight. Polymerized samples were cut into ultrathin sections (60–70 nm), placed on carbon-coated copper grids, and negatively stained with uranyl acetate and lead citrate before examination in a Philips CM10 TEM transmission electron microscope (Philips Electron Optics, Eindhoven, The Netherland).

TEM images were analysed with ImageJ Java 8 software (National Institute of Health, Bethesda, MD, USA). For each growth condition, the diameter of cells and the cell wall thickness of the six largest cells for each cutting section was measured. For each cell, cell wall thickness was measured in three places at the widest, at the narrowest, and at an additional random spot. Cell wall thickness measurements were taken at ×46,000 magnification.

### 4.6. Peptidoglycan Measurement

#### 4.6.1. Production of the Standard Curve

**Figure 8:**
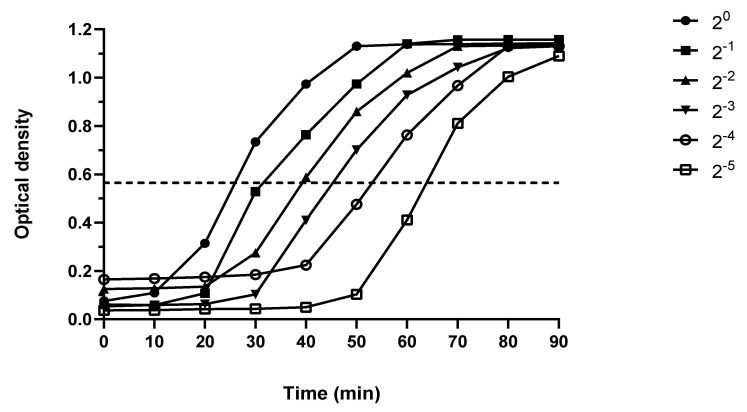
Kinetics of the absorption changes observed at a wavelength of 650 nm to determine the midpoint of the sigmoidal curve as marked by the dotted line (=OD 0.57).

**Figure 9:**
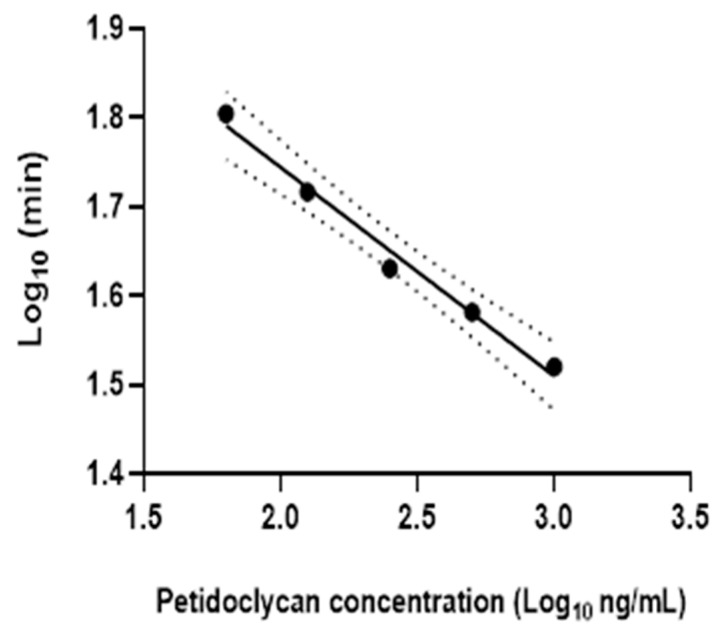
Standard curve for peptidoglycan assay. The y axis is the time (Log_10_ min) till the diluted standard reached the mid-point of the sigmoidal curve, plotted against the log of the concentration of peptidoglycan in that standard (PGN; Log_10_ ng/mL).

The manufacturer’s standard of *Micrococcus luteus* peptidoglycan was subjected to serial 10-fold dilutions in PBS and then mixed with an equal volume of Silkworm Larva Plasma solution and incubated at 30 °C, and the kinetics of absorption at 650 nm was observed every 10 min for >90 min using a PHERAstar FS microplate reader (BMG Labtech, Ortenberg, Germany). The mid-point of the standard’s sigmoidal curves is shown in Figure 8 and was equal to an optical density of 0.57. The log_10_ of the time taken to reach the midpoint, i.e., an optical density of 0.57 for each standard, was plotted against the known concentration of peptidoglycan (log10) in the diluted standards, as shown in Figure 9.

#### 4.6.2. Measurement of Peptidoglycan

Bacterial protein was extracted according to our published methods [30,31]. The concentration of extracted protein was quantified by bicinchoninic acid (BCA) assay (PierceTM BCA protein kit assay, Thermo Fisher Scientific) 562 nm wavelength (PHERAstar FS, BMG Labtech, Ortenberg, Germany), as per the manufacturer’s instructions.

The amount of peptidoglycan in 5 µg of extracted protein from planktonic, hydrated biofilm, and DSB was determined in triplicate. The extracted protein in 50 µL PBS was mixed with equal volume of Silkworm Larva Plasma solution and incubated at 30 °C, and the kinetics of absorption at 650 nm was observed every 10 min for >90 min, as above. The amount of peptidoglycan was determined from a standard curve using peptidoglycan from *Micrococcus luteus*, as per the manufacturer’s instructions.

### 4.7. Disinfectant Efficacy Testing

Disinfectant tolerance of approximately 10^7^ planktonic and biofilm *S. aureus* was determined by diluting sodium hypochlorite to available chlorine between 50 and 5000 mg/L. The amount of free chlorine was verified by iodometric titration.

Disinfectant efficacy was tested by adding a biofilm-covered coupon to 2 mL of disinfectant and reacting for 5 min. The coupons were then rinsed twice in PBS before being placed in 2 mL of 20% bovine serum albumin to inactivate the disinfectant action.

Residual CFU determined following sonication and plating as described above. The efficacy of sodium hypochlorite against planktonic organisms was determined by adding 10 µL of 24 h bacterial culture in TSB containing 10^7^ *S. aureus* to 2 mL of disinfectant, reacting for 5 min before adding neutralising broth, serial dilutions, and culture. The efficacy of each disinfectant concentration was tested four times and was tested in the absence of added soil or hard water.

Disinfectant efficacy testing controls were subjected to the same protocol and included: positive controls—water replaced the disinfectant; negative controls—media replaced the bacteria in the planktonic test, or a sterile coupon was used instead of a biofilm covered coupon for the biofilm tests; neutraliser toxicity control—bacteria were reacted with neutralising broth only, and CFU compared to the positive control; and, finally, neutraliser control—disinfectant and neutralising broth were mixed, and then bacteria were immediately added and CFU compared to positive control.

### 4.8. Data Analysis

Data analysis for TEM cell width and peptidoglycan measurements and comparison of cell numbers were performed using a one-way ANOVA with Tukey’s multiple pairwise comparisons to determine the significance of differences. A one-way ANOVA with Dunnets multiple comparisons versus the control group was used to check for significant differences in maximum cell size between one-day planktonic and biofilm cells. Normality was checked using the Shapiro–Wilks test and equal variance using the Brown–Forsythe test. The results are displayed as mean values plus or minus the standard deviation.

## 5. Conclusions

Peptidoglycan synthesis is upregulated in *S. aureus* biofilm and results in increased cell wall thickness and increased deposition of EPS. The cell wall becomes thicker as the biofilm ages, or if the biofilm is subjected to periodic osmotic stress. In addition, D-alanine transfer protein DltB, which aids the D-alanine modification of teichoic acid, is upregulated. These cell wall changes mimic those found in daptomycin-resistant *S. aureus* 24 due to genetic mutation and may contribute to the antibiotic tolerance seen in *S. aureus* biofilms lacking genetic mutations. The tolerance of *S. aureus* to sodium hypochlorite was increased as peptidoglycan synthesis increased, which was reflected in increased cell wall width and increased EPS production. Pathways and mechanisms leading to clinical DSB formation are unknown, but, still, our findings may trigger target-based approaches for designing novel chemical classes aimed at peptidoglycan synthesis to aid in the killing of biofilms.

Treatment of biofilm infections is problematic, particularly if a foreign body such as an implantable device is present, as biofilm infections are generally tolerant of antibiotic therapy. Mechanisms that have been proposed include the inactivation of antibiotics by the EPS, increased efflux pumps, quorum sensing, and persister cells. In this study, we have shown that as the biofilm ages, the cell wall becomes thicker. We suggest that the cell wall phenotypic changes associated with biofilm formation is one of the major contributors to antibiotic tolerance of biofilms. The development of new antibiotics could focus on the cell wall or molecules that can bypass the cell wall.

DSB contaminate over 90% of hospital surfaces and can contain multidrug-resistant organisms such as MRSA. Collaborate laboratory research showed that DSB are more tolerant of disinfectants and harder to physically remove than planktonic organisms and even hydrated biofilm. Current methods of cleaning and decontamination are not sufficient to kill or remove DSB. The presence of DSB provides a source of pathogenic bacteria for transmission within healthcare. In this study, we have demonstrated thickened bacterial cell wall in DSB to reduce water loss from the cell in addition to the thickened EPS in DSB. Determining the mechanism behind DSB tolerance can lead to the development of better cleaning agents against DSB.

## Data Availability

Proteome data generated during and/or analysed during the current study are available in the ProteomeXchange repository, with identifier PXD033499. The other datasets generated and/or analysed during the current study are available from the corresponding author on reasonable request.

## Supplementary Materials

The following supporting information can be downloaded at: https://www.mdpi.com/article/10.3390/ijms24054983/s1.
