# Supplementary material for: Staphylococcus aureus Cell Wall Phenotypic Changes Associated with Biofilm Maturation and Water Availability: A Key Contributing Factor for Chlorine Resistance
Source: Int J Mol Sci. 2023 Mar 5;24(5):4983. doi: 10.3390/ijms24054983 (PMC10003762; doi:10.3390/ijms24054983)
Supplement: Supplementary file 1 [file ijms-24-04983-s001.zip › ijms-2233685-supplementary.pdf]

Supplementary Methods: Proteomics analysis of biofilm

Protein Extraction and Fractionation

Planktonic bacteria were pooled from three independent growth of 24 h *Staphylococcus aureus* cultures and were mixed with lysis buffer containing 100 mM Triethylammonium bicarbonate (TEAB; Sigma-Aldrich, St. Louis, MO, USA) pH 8.5 and 1% (*w/v*) sodium deoxycholate (Sigma-Aldrich) at 10:1 ratio (supernatant: lysis buffer), while *S. aureus* biofilms (3-day biofilm, 12-day biofilm and DSB) coated coupons (n = 24) were washed to remove non-adherent cells and coupons individually placed in 2 mL of phosphate-buffered saline (PBS) and lysis buffer and incubated overnight with gentle shaking at 4°C. Samples were probe sonicated in an ice-cold environment (Sonic Ruptor, Omni International, Kennesaw, Georgia, GA, USA) for 2 min at 50% power and 70% pulses. The samples were centrifuged at 12,000× *g* for 10 min and the supernatant was filtered through a 10 kDa molecular weight cut-off (MWCO) ultra-membrane filter tube (Sigma-Aldrich) before centrifugation at 4000× *g* for 20 min. Protein samples were washed three times with PBS to remove TSB and lysis buffer and further concentrated using a 3 KDa MWCO filter tube (Sigma Aldrich).

The BCA protein assay (Thermo Fisher Scientific, Waltham, MA, USA) was used to measure protein concentration at 562 nm wavelength as per the manufacturer's instructions.

Protein Reduction, Alkylation, and Digestion

A total of 40 µg protein of each biological replicate was reduced with 5 mM DTT for 15 min at RT and alkylated with 10 mM iodoacetamide (IAA) in the dark for 30 min at RT. Alkylated samples were diluted with 100 mM TEAB pH 8.5. Samples were digested overnight at RT by the addition of Lys-C at a ratio of 1:30 followed by the addition of trypsin at a ratio of 1:30 for 5.5 h at 37°C. Samples were adjusted to 1% (*v/v*) TFA, and the precipitated deoxycholate was removed by centrifugation. Samples were centrifuged at 14,100× *g* and desalted with 0.2% TFA washing using SDB-RPS (3M-Empore) Stage Tips (Thermo Fisher Scientific). Samples were eluted with 5% ammonium hydroxide in 80% acetone and centrifuged at 1000× *g* for 5 min, vacuum dried and stored at -20°C until further processing.

TMT Labelling and High pH Fractionation

TMT10plex™ Isobaric Label Reagent Set (Thermo Fisher Scientific) was used. TMT reagents (0.8 mg) were dissolved in acetone (85 µL) of which 41 µL was added to the reconstituted (100 µL of 100 mM TEAB pH 8.5) samples and incubated for 1 h at RT. A volume of 8 µL of 5% hydroxylamine was added to each TMT-labelled sample and incubated for 15 min at RT. A volume of 2 µL of each labelled sample was pooled and vacuum dried and reconstituted in 30 µL 0.1% formic acid (FA) (Merck, Kenilworth, NJ, USA) solution, centrifuged for 5 min at 14,000× *g* and analysed with mass spectrometer (for detailed information see nanoLC-ESI-MS/MS using Orbitrap Elite).

Data searching was conducted using Proteome Discoverer 1.3 (for detailed information see data processing). Based on the applied normalization values from this search result an equal number of peptides were taken from each sample, pooled and vacuum dried (miVac). The dried labeled sample was resuspended in buffer A (5 mM ammonia, pH 10.5) and fractionated by high pH reverse phase-high pressure liquid chromatography (RP-HPLC; Agilent Technologies, Santa Clara, CA, USA). The dried labeled sample was resuspended in buffer A. After sample loading and washing with 97% buffer A for 10 min, the concentration of buffer B (5 mM ammonia solution with 90% Acetone, pH 10.5) was increased from 3% to 30% for 55 min; 70% for 10 min; 90% for 5 min at a flow rate of 300 µL/min. The eluent was collected every 2 min at the beginning until 16 min and every 1 min intervals for the remainder of the gradient. The fractionated sample was pooled into 19 fractions and dried in miVac. Finally, each fraction was resuspended in 55 µL of 0.1% FA for mass spectrometer (MS) analysis.

Nanoflow LC-ESI-MS/MS

All samples were run on two sequential mass spectrometer systems Orbitrap Elite (Thermo Fisher Scientific) and Q Exactive (Thermo Fisher Scientific).

<u>Nanoflow LC-ESI-MS/MS Using Orbitrap Elite</u>

An Orbitrap Elite (Thermo Fisher Scientific) mass spectrometer equipped with PicoView 550 Nanospray Source (New Objectives), an Eksigent ultra-pressure liquid chromatography (UPLC) system (AB SCIEX) consisting of an ekspert™ nanoLC 425 UPLC pump and ekspert™ nanoLC 400 (Thermo Fisher Scientific) autosampler was used for acquiring data. 20 µL of each fraction was loaded onto a self-packed 100 µm × 3.5 cm trap column with Halo® 2.7 µm 160 Å ES-C18 (Advanced Materials Technology, Wilmington, DE, USA) and desalted with loading buffer [0.1% FA] at a flow rate of 4 µL/min for 10 min. Peptides were eluted onto a self-packed analytical column 100 µm × 30 cm with Halo® 2.7 µm 160 Å ES-C18 (Advanced Materials Technology) with the linear gradients of mobile phase A (0.1% FA/5% DMSO) and mobile phase B (0.1% FA/5% DMSO) starting with B (1–10%) for 0.1 min, B (10–20%) for 52 min, B (20–32%) for 48 min followed by (32–43%) for 20 min with a flow rate of 450 nL/min across the gradient. The eluent from the trap was diluted with 100 nL/min of buffer A before

reaching the analytical column. The peptides refocused and separated over the analytical column at 60°C. Peptides were ionized by electrospray ionization, and data-dependent MS/MS acquisition was carried out using an Orbitrap Elite (Thermo Fisher Scientific) consisting of 1 full MS1 (R = 120 K) scan acquisition from 380 to 1600 *m*/*z*, and 15 higher energy collisional dissociation (HCD) type MS2 scans (R = 30 K).

Nanoflow LC-ESI-MS/MS Using Q Exactive

A Q Exactive (Thermo Fisher Scientific) Mass Spectrometer equipped with Nano spray Source and Easy nLC 1000 (Thermo Fisher Scientific) was used for acquiring data. 10 µL of each fraction was loaded onto a self-packed 100 µm × 3.5 cm reversed-phase peptide trap with Halo® 2.7 µm 160 Å ES-C18 (Advanced Materials Technology) desalted with 20 µL of loading buffer (0.1% FA) and the peptide trap was then switched on-line with the analytical column a self-packed 75 µm × 3.5 cm Halo® 2.7 µm 160 Å ES-C18 column. Peptides were eluted with the linear gradients of mobile phase A (0.1% FA) and buffer B [100%(*v/v*) Acetone, 0.1%(*v/v*) FA] starting with (1–30%) for 110 min, B (30–85%) for 2 min followed by 85% B for 8 min with a flow rate of 300 nL/min. Peptides were ionized by electrospray ionization and data-dependent MS/MS acquisition was carried out using a Q-Exactive consisting of 1 full MS1 (R = 70 K) scan acquisition from 350 to 1850 m/z, and 10 HCD type MS2 scans (R = 70 K).

Database Search, Statistical Analysis, and Bioinformatics

Database search, statistical analysis, and bioinformatics were performed as below. The raw data files were submitted to Proteome Discoverer (version 1.3, Thermo Fisher Scientific). The data were processed using Sequest and Mascot (Matrix Science, London, UK) against the *S. aureus* reference strain (ATCC 25923) from Genbank CP009361 and CP009362. For protein identification, the following options were used: peptide mass tolerance = 10 ppm; MS/MS tolerance = 0.1 Da; enzyme = trypsin, missed cleavage = 1; fixed modification, carbamidomethyl (C), TMT10-plex (K) and TMT10-plex (N-term); variable modification, oxidation (M), Deamidated (N, Q) and Acetyl (N-Terminus). Quantification was performed based on the peak intensities of reporter ions in the MS/MS spectra. A below 1% false discovery rate was selected as the cut-off for peptide identification. Protein quantification was based on the total intensity of the assigned peptides. After the extraction of protein ratios with Proteome Discoverer, additional processing and statistical analyses were performed using the TMTPrePro R package. BLAST search was performed using highly annotated strains *S. aureus* N315 and *S. aureus* COL. Proteins were considered upregulated when the TMT ratio was above 1.5 and downregulated when the TMT ratio was lower than 0.66 in biofilm growth compared to planktonic growth with a significant p-value < 0.05. Significant differentially expressed proteins (>2-fold) were determined by using VENNY (v.2.1) (http://bioinfogp.cnb.csic.es/tools/venny/, accessed on 25 June 2018) and processed further to gain more functional insights. Metabolic pathways of identified proteins were analysed by using the Kyoto Encyclopedia of

Genes and Genomes (KEGG) mapper (https://www.genome.jp/kegg/tool/map_pathway2.html, accessed on 6 August 2018). Subcellular localisation of identified proteins was analysed by using PSORTb (version 3.0.2) (http://www.psort.org/psortb/index.html, accessed on 23 January 2018). The protein-protein interaction (PPI) network of significantly differentially expressed proteins was analysed by STRING software v.10.0 (http://string-db.org/, accessed on 25 June 2018).

Supplementary Tables

Table S1. Functional classification of significantly upregulated proteins (> 2-fold) common in biofilm compared to planktonic culture (P<0.05).

Protein Pathways	Accession ID	Protein Name	Gene Name	Fold change			Subcellular Localisation
				3-day	12-day	DSB	
Purine metabolism	AIO20958.1	guanosine 5'-monophosphate oxidoreductase	guaC SACOL1371	2.28	3.28	2.97	Cytoplasmic
	AIO21316.1	hypothetical protein KQ76_08470	SAS049	3.38	2.21	2.21	Unknown
	AIO21769.1	hypothetical protein KQ76_11075	SA1933	2.47	2.10	2.26	Cytoplasmic
	AIO20968.1	exonuclease	sbcD SA1180	2.40	2.36	2.73	Cytoplasmic
Metabolic pathways, Biosynthesis of secondary metabolites, Biosynthesis of antibiotics, Propanoate metabolism, Valine, leucine and isoleucine degradation	AIO21211.1	2-oxoisovalerate dehydrogenase	bfmBAA	3.19	2.12	2.06	Cytoplasmic
	AIO20399.1	membrane protein	SACOL0790	4.75	3.30	3.28	CM
	AIO21578.1	membrane protein	SACOL1947	16.46	3.88	2.99	CM
Cationic antimicrobial peptide (CAMP) resistance, *Staphylococcus aureus* infection, Two-component system	AIO20508.1	D-alanine transfer protein DltB	DltB	2.10	3.02	3.20	CM

Table S2. Functional classification of significantly downregulated proteins (> 2-fold) common in biofilm compared to planktonic culture (P<0.05).

Protein Pathways	Accession ID	Protein Name	Gene Name	Fold change			Subcellular Localisation
				3-day	12-day	DSB	
	AIO20660.1	chitinase	SA0914	0.12	0.09	0.06	Unknown
Metabolic pathways, Glycerolipid metabolism	AIO19987.1	lipase	lip2 geh SA0309	0.24	0.14	0.10	Extracellular
	AIO20583.1	hypothetical protein KQ76_04610	SACOL1009	0.51	0.46	0.35	CM
	AIO21549.1	Fur family transcriptional regulator	perR SACOL1919	0.25	0.48	0.42	Cytoplasmic
	AIO20100.1	hypothetical protein KQ76_01960	SA0395	0.35	0.14	0.12	Unknown
	AIO22290.1	N-acetylmuramoyl-L-alanine amidase	SACOL2666	0.17	0.14	0.12	Extracellular
Base excision repair	AIO21349.1	DNA-3-methyladenine glycosylase	tag	0.14	0.40	0.36	Unknown
Metabolic pathways, Glycerolipid metabolism	AIO22317.1	lipase	lip1 SA2463	0.35	0.20	0.19	Extracellular
	AIO22350.1	peptidase		0.28	0.13	0.12	Extracellular
Two-component system	AIO21182.1	transcriptional regulator	srrA SA1323	0.28	0.33	0.32	Cytoplasmic
Metabolic pathways, Aminoacyl-tRNA biosynthesis	AIO20194.1	glutamyl-tRNA synthetase	gltX SA0486	0.47	0.39	0.39	Cytoplasmic
	AIO21805.1	uridylyltransferase	SA1974	0.28	0.36	0.36	Cytoplasmic
	AIO19900.1	nitric oxide dioxygenase	SA0231	0.47	0.34	0.35	Cytoplasmic
	AIO21508.1	serine protease	splE SACOL1865	0.24	0.40	0.41	Extracellular
	OOC90902.1	calcium-binding protein	SACOL1846	0.08	0.05	0.05	Unknown
RNA degradation	AIO21274.1	molecular chaperone DnaK	dnaK SA1409	0.37	0.43	0.47	Cytoplasmic
Aminoacyl-tRNA biosynthesis	AIO20737.1	phenylalanine--tRNA ligase	pheS SA0985	0.31	0.35	0.39	Cytoplasmic
	AIO21788.1	HAD family hydrolase	SA1957	0.38	0.30	0.34	Cytoplasmic
	AIO20874.1	50S ribosomal protein L7	SA1111	0.27	0.34	0.38	Unknown
	AIO21842.1	toxin	SACOL2197	0.32	0.40	0.46	CM
Bacterial invasion of epithelial cells	AIO22136.1	fibronectin-binding protein	fnbA SA2291	0.39	0.23	0.26	Cellwall
Ribosome	AIO21871.1	30S ribosomal protein S17	rpsQ SACOL2230	0.21	0.04	0.04	Cytoplasmic
	AIO22096.1	chloramphenicol resistance protein DHA1	SA2241	0.31	0.21	0.18	CM

	OOC94196.1	hypothetical protein BWO94_03305		0.17	0.13	0.07	Unknown
	AIO20603.1	2', 3'-cyclic nucleotide 2'-phosphodiesterase	SACOL1031	0.28	0.18	0.17	Cytoplasmic
	AIO22141.1	MerR family transcriptional regulator	SACOL2517	0.28	0.39	0.51	Unknown
Metabolic pathways, Folate biosynthesis	AIO20380.1	7-cyano-7-deazaguanine synthase	queC SA0667	0.34	0.50	0.48	Cytoplasmic
	AIO19755.1	ATPase AAA		0.30	0.27	0.21	CM
Staphylococcus aureus infection	AIO20758.1	fibrinogen-binding protein	fib efb SA1003	0.47	0.27	0.29	Extracellular
	AIO21801.1	multidrug MFS transporter	SA1970	0.44	0.24	0.17	CM

Table S3. Functional classification of significantly upregulated proteins (> 2-fold) common in 3-day and 12-day hydrated biofilm compared to planktonic culture (P<0.05).

Protein Pathways	Accession ID	Protein Name	Gene Name	Fold Change		Subcellular Localisation
				3-day	12-day	
	AIO20486.1	UPF0337 protein SA0772	SA0772	5.09	2.36	Unknown
	AIO21982.1	Uncharacterized protein	SACOL2344	3.3	2.32	Cytoplasmic
	AIO21786.1	Lytic regulatory protein truncated with Tn554	truncated-SA	2.89	2.02	CM
Metabolic pathways, Amino sugar and nucleotide sugar metabolism, Biosynthesis of antibiotics, Alanine, aspartate and glutamate metabolism	AIO21790.1	Glutamine--fructose-6-phosphate aminotransferase [isomerizing] (EC 2.6.1.16)	glmS SACOL2145	2.37	2.13	Cytoplasmic

Table S4. Functional classification of significantly upregulated proteins (> 2-fold) common in 12-day biofilm and DSB compared to planktonic culture (P<0.05).

Protein Pathways	Accession ID	Protein Name	Gene Name	Fold Change		Subcellular Localisation
				12-day	DSB	
	AIO22305.1	Uncharacterized protein	SA2451	5.52	4.40	Unknown
	AIO22223.1	Uncharacterized protein	SA2371	3.59	2.46	Unknown
Metabolic pathways, Biosynthesis of secondary metabolites, Propanoate metabolism, Valine, leucine and isoleucine degradation	AIO21210.1	2-oxoisovalerate dehydrogenase, E1 component, beta subunit (EC 1.2.4.1)	SACOL1561	3.11	3.36	Cytoplasmic
	AIO20294.1	Na+/H+ antiporter, putative	SACOL0687	2.98	3.04	Unknown
	AIO20937.1	Uncharacterized protein	SAS040	2.84	3.94	Unknown
	AIO21539.1	Uncharacterized protein	SA1668	2.50	2.58	Cytoplasmic
Butanoate metabolism	AIO19795.1	Diacetyl reductase [(S)-acetoin forming] (EC 1.1.1.304) (Acetoin(diacetyl) reductase) (AR) (Meso-2,3-butanediol dehydrogenase)	butA SA0122	2.49	2.35	Cytoplasmic
Metabolic pathways, Pyrimidine metabolism, Alanine, aspartate and glutamate metabolism	AIO20804.1	Carbamoyl-phosphate synthase small chain (EC 6.3.5.5) (Carbamoyl-phosphate synthetase glutamine chain)	carA pyrAA SA1045	2.41	2.38	
Pantothenate and CoA biosynthesis	AIO21707.1	Holo-[acyl-carrier-protein] synthase (Holo-ACP synthase) (EC 2.7.8.7) (4'-phosphopantetheinyl transferase AcpS)	acpS dpj SA1875	2.37	3.18	
	AIO20325.1	Uncharacterized protein	SACOL0715	2.35	2.18	
Ribosome	AIO21864.1	50S ribosomal protein L18	rplR SA2032	2.33	2.19	Cytoplasmic
Metabolic pathways, Pyrimidine metabolism	AIO21765.1	CTP synthase (EC 6.3.4.2) (Cytidine 5'-triphosphate synthase) (Cytidine triphosphate synthetase) (CTP synthetase) (CTPS) (UTP--ammonia ligase)	pyrG ctrA SA1929	2.32	2.24	Cytoplasmic

Metabolic pathways, Phosphotransferase system, Galactose metabolism	AIO21825.1	PTS system lactose-specific EIICB component (EIICB-Lac) (EII-Lac) [Includes: PTS system lactose-specific EIIC component (Lactose permease IIC component); PTS system lactose-specific EIIB component (EC 2.7.1.207) (Lactose-specific phosphotransferase enzyme IIB component)]	lacE SACOL2181	2.27	2.54	CM
	AIO20268.1	Uncharacterized protein	SA0559	2.25	3.02	Unknown
	AIO20620.1	Uncharacterized protein	SACOL1585	2.20	3.15	Unknown
	AIO19785.1	Putative ornithine cyclodeaminase	sbnB	2.20	3.61	
Ribosome	AIO21878.1	50S ribosomal protein L23	rplW SA2045	2.15	2.41	Cytoplasmic
Ribosome	AIO21848.1	50S ribosomal protein L13	rplM SA2017	2.14	3.28	Unknown
Metabolic pathways, Pyrimidine metabolism	AIO20807.1	Orotate phosphoribosyltransferase (OPRT) (OPRTase) (EC 2.4.2.10)	pyrE SA1048	2.13	2.30	Cytoplasmic
Ribosome	AIO21879.1	50S ribosomal protein L4	rplD SA2046	2.03	2.33	Unknown
	AIO20666.1	Phosphoribosylformylglycinamidine synthase subunit PurS (FGAM synthase) (EC 6.3.5.3) (Formylglycinamide ribonucleotide amidotransferase subunit III) (FGAR amidotransferase III) (FGAR-AT III) (Phosphoribosylformylglycinamidine synthase subunit III)	purS SA0919	2.01	2.09	
	OOC94735.1	hypothetical protein BWO94_01050		3.63	4.91	Unknown

Table S5. Functional classification of significantly downregulated proteins (> 2-fold) common in 3-day and 12-day hydrated biofilm compared to planktonic culture (P<0.05).

Protein Pathways	Accession ID	Protein Name	Gene Name	Fold Change		Subcellular Localisation
				3-day	12-day	
	AIO20515.1	hypothetical protein KQ76_04260	SACOL0943	0.33	0.50	
	AIO21565.1	hypothetical protein KQ76_09970	SAS054	0.32	0.37	

Table S6. Functional classification of significantly downregulated proteins (> 2-fold) common in 12-day biofilm and DSB compared to planktonic culture (P<0.05).

Protein Pathways	Accession ID	Protein Name	Gene Name	Fold Change		Subcellular Localisation
				12-day	DSB	
	AIO20349.1	hypothetical protein KQ76_03365	SACOL0741	0.30	0.25	Cytoplasmic
Staphylococcus aureus infection	AAX11326.1	Enterotoxin type G (SEG)	entG seg SA1642	0.40	0.41	Extracellular
	AIO20626.1	Uncharacterized protein	SACOL1579	0.27	0.25	CM
	AIO20189.1	excinuclease ABC subunit B	SA0481	0.36	0.40	Cytoplasmic
	AIO20628.1	Uncharacterized protein	SACOL1577	0.46	0.50	CM
	AIO22180.1	SA2330 protein	SA2330	0.34	0.32	Cytoplasmic
Metabolic pathways, Galactose metabolism, Phosphotransferase system (PTS)	AIO19907.1	PTS sugar transporter subunit IIA	SA0236	0.24	0.29	Cytoplasmic
	AIO20451.1	SA0734 protein	SA0734	0.45	0.35	Cytoplasmic
	AIO21128.1	hypothetical protein KQ76_07495		0.35	0.28	Cytoplasmic
	AIO20578.1	Competence protein, putative	SACOL1004	0.32	0.29	Cytoplasmic
	AIO20988.1	SA1196 protein	SA1196	0.23	0.27	Cytoplasmic
Metabolic pathways, Microbial metabolism in diverse environments, Purine metabolism, Arginine biosynthesis	AIO21919.1	urease subunit gamma	ureA SA2082	0.35	0.45	Cytoplasmic
Metabolic pathways, Purine metabolism, Pyrimidine metabolism	AIO20222.1	deoxycytidine kinase	SA0514	0.43	0.50	Cytoplasmic
	AIO21618.1	hypothetical protein KQ76_10250	SAS056	0.46	0.44	
Metabolic pathways, DNA replication, Pyrimidine metabolism, Purine metabolism, Homologous recombination, Mismatch repair	AIO20145.1	DNA polymerase III subunit delta'	SACOL0526	0.31	0.34	Cytoplasmic

	AIO21816.1	Uncharacterized protein	SA1983	0.17	0.25	Cytoplasmic
	AIO21983.1	esterase	SA2140	0.48	0.44	Cytoplasmic
	AIO22015.1	SA2166 protein	SA2166	0.43	0.28	CM
	AIO20581.1	Uncharacterized protein	SA0861	0.38	0.39	CM
	AIO22174.1	thioredoxin	SA2324	0.42	0.47	Cytoplasmic
Two-component system	AIO21715.1	Sensor protein KdpD	kdpD	0.39	0.40	CM
	AIO20099.1	Uncharacterized protein	SA0394	0.30	0.25	Unknown
Staphylococcus aureus infection	AIO21515.1	Staphylococcal enterotoxin B	seb SACOL0907	0.34	0.23	Extracellular
Ribosome	AIO21867.1	30S ribosomal protein S14	rpsZ rpsN1 SA2034.1 SAS079	0.26	0.29	Cytoplasmic

Table S7. Sodium hypochlorite efficacy testing against *S. aureus* in planktonic and three different biofilm growth mode.

	Chlorine (mg/L)[#]	**Residual** CFU (Log10)	**STDEV**	**Average** Log reduction	**STDEV**
Planktonic	500	0	0	6.958	0.0
	250	3.117	0.037	3.841	0.037
	100	3.825	0.056	3.133	0.056
	50	4.929	0.042	2.029	0.042
3-day biofilm	1000	0	0	7.161	0.0
	500	4.043	0.139	3.117	0.139
	250	4.698	0.014	2.462	0.014
	100	5.801	0.108	1.359	0.108
12-day biofilm	2000	0.000	0.000	7.233	0.0
	1000	3.406	0.054	3.825	0.054
	500	4.673	0.053	2.558	0.053
	250	5.539	0.030	1.692	0.030
12-day DSB	5000	0.000	0.000	7.052	0.0
	2000	4.209	0.130	2.842	0.130
	1000	4.744	0.018	2.307	0.018
	500	5.516	0.022	1.535	0.022

[#] Disinfectant contact time was 5 min, disinfectant concentration is expressed as free chlorine levels (mg/L).
